# Robot-Assisted Rehabilitation as a Form of Progressive Therapy in Upper Extremity Motor Recovery After Stroke: A Systematic Review

**DOI:** 10.3390/jcm15082951

**Published:** 2026-04-13

**Authors:** Wiktoria Załoga, Paulina Magdalena Ostrowska, Rita Hansdorfer-Korzon

**Affiliations:** Department of Physiotherapy, Medical University of Gdansk, 7 Dębinki Street, 80-211 Gdańsk, Poland; wiktoria.zaloga@gumed.edu.pl (W.Z.); rita.korzon@gumed.edu.pl (R.H.-K.)

**Keywords:** stroke, robot-assisted rehabilitation, upper extremity, motor recovery

## Abstract

**Background**: Statistics show that the number of patients suffering from stroke and living with disability as a result has increased significantly worldwide between 1990 and 2021. This implies the necessity of continuous improvement of both treatment methods and rehabilitation, which is essential in the treatment process. According to the International Classification of Functioning (ICF), as many as 88% of patients do not regain functionality in their affected upper limbs six months after a vascular incident, which negatively affects their quality of life. Upper limb rehabilitation using robots is a progressive approach to restoring not only lost limb function, but above all, independence in activities of daily living. **Aim of the study**: The aim of this review is to determine the actual effects of rehabilitation using various robots in patients after cerebrovascular accidents and to compare these effects with those achieved by patients who participated in a non-robotic rehabilitation program. **Methods**: Studies published between 2019 and 2025 were included in the analysis. The analysis was written according to the Preferred Reporting Items for Systematic Reviews and Meta-Analyses (PRISMA) checklist. **Results**: Studies have shown improvement in upper limb function in patients exercising both with and without the use of a robot, but statistical data clearly show better results in patients undergoing therapy with robots. **Conclusions**: Studies have shown that the use of a rehabilitation program involving robots brings measurable improvements in many different aspects of upper limb function, and evidence confirming the effectiveness of this therapy encourages further research, refinement, and dissemination of this method and technology.

## 1. Introduction

Each year, approximately 12 million people worldwide suffer a stroke, and a substantial proportion of survivors are left with permanent disability [1,2]. It is estimated that over the last three decades, the number of patients dying from stroke has increased almost threefold, and it is currently the third leading cause of death and disability (combined) according to the DALY index (over 160 million DALYs worldwide). The estimated cost of stroke is currently around US$890 billion per year (0.66% of global GDP) and is expected to double by 2050 [1].

In Poland as well, stroke is one of the most serious public health challenges. According to data from the National Health Fund and system reports, the number of ischemic strokes in Poland is approximately 73,000–75,000 per year, accounting for about 87–89% of all stroke cases [3]. Stroke is both the second leading cause of death and the leading cause of permanent disability in the adult population [4].

A key component of post-stroke treatment is neurological rehabilitation, the early initiation of which significantly impacts patients’ functional prognosis. According to the latest systemic data, in 2023, only 22.4% of stroke patients in Poland received neurological rehabilitation within 14 days of hospital discharge. In absolute terms, this amounts to approximately 14,300 patients annually [3,4]. These data highlight a significant systemic gap, as experts estimate that 30–40% of patients require comprehensive rehabilitation. This means that the healthcare system in Poland meets only about half of the population’s actual needs [4]. Furthermore, in some regions, only about 14% of patients are admitted to neurological rehabilitation units, and the waiting time for services can be as long as 16 months [4]. This problem is structural in nature and has persisted for many years—earlier analyses have already indicated that the available rehabilitation infrastructure covers only about 20–25% of the demand for such services [5].

Despite a significant increase in funding for post-stroke rehabilitation—from approximately PLN 165 million in 2018 to over PLN 450 million in 2022–2023—no corresponding improvement in the availability of services has been observed [6]. This suggests the existence of systemic barriers, such as a limited number of neurological rehabilitation units, staff shortages, and a lack of effective coordination of post-stroke care.

Stroke is a vascular disease that leads to damage to the central nervous system. Patients who have survived a vascular incident present with characteristic clinical symptoms, either local or more global. The primary disorder that patients face is weakness on one side of the body (hemiplegia/hemiparesis), which can affect both the upper and lower limbs. It is estimated that approximately 80% of patients experience motor system disabilities such as loss of muscle mass or coordination. Maintaining postural control in the trunk, maintaining balance in both higher and lower positions, and gait patterns become very difficult for most patients [7].

Rehabilitation of the upper extremity directly affected seems to be the greatest challenge for both patients and physical therapists [8]. Dysfunctions manifesting as paresis and overlapping superficial and deep sensory disturbances affect up to 50–65% of patients. Patients have difficulty understanding the position of their own body in space, and their perception of mechanical stimuli is weakened or completely eliminated, which is the cause of ineffective motor recovery and delays the effects of physical therapy [9].

The statistics show that only 5–20% percent of post-stroke patients fully recover upper limb function, with almost half of them developing spasticity later on [8]. It is also estimated that only about twenty percent of stroke survivors undergoing rehabilitation regain full recovery and motor function of their upper limbs. They become dependent in matters of managing basic daily living activities and just essentially supporting themselves [10,11]. Their disability—as in hemiparesis—requires constant therapy and medical attention. Therefore, it affects their quality of life, social participation, and often their mental state directly [8,12,13].

Robot-assisted therapy is a newly researched form of physical therapy after stroke, which focuses on regaining motor function of the impaired limb [11]. During the past twenty years, many robot appliances were both developed and assessed, providing outcomes equivalent to dose-matched conventional therapy [14,15,16]. Robot -assisted therapy is designed to enhance the functional capacity as well as sensory and cognitive performance [15,17].

Robots enable training of both proximal and distal movements of the upper limb. In the shoulder and elbow joints, the training includes flexion, extension, abduction, and adduction, while in the distal segments of the limb, exercises involving grasping, releasing, and manipulating objects are possible. Of particular importance is the ability to perform functional movements, such as reaching or transferring objects, which form the basis of task-oriented training [18].

Studies have also shown that robotic therapy leads to improved motor control and muscle strength, particularly in patients with moderate motor deficits and in the early post-stroke phase [19]. In cases of flaccid paresis, robots are particularly useful because they enable movement initiation through “assist-as-needed” modes and limb guidance, which allows for the activation of patients without active muscle activity [18].

With regard to sensory disorders, available studies indicate that robotic upper limb rehabilitation may lead to improvements in proprioception and sensory integration. A randomized clinical trial (Farrens et al., 2025) has shown that training tailored to sensory deficits can improve deep sensation and hand function, suggesting a potential role for robots not only in motor rehabilitation but also in sensory rehabilitation [20].

The use of robots in the treatment of spasticity, however, remains limited. A systematic review (Bertani et al., 2017) indicates that robotic therapy does not have a significant direct effect on reducing spasticity, although secondary improvement resulting from increased physical activity is possible [21].

On the other hand, a problem is the high degree of heterogeneity among studies, resulting from differences in device design (exoskeletons vs. end-effector robots), the number of degrees of freedom, treatment intensity, and patient eligibility criteria. These factors make it difficult to unequivocally assess efficacy and develop clinical practice guidelines [22].

## 2. Materials and Methods

This systematic review was conducted and reported in accordance with the PRISMA guidelines [23]. The completed PRISMA 27-item reporting checklist has been included as a Supplementary File to ensure transparency and completeness of reporting. The review protocol was not prospectively registered in PROSPERO.

### 2.1. Eligibility Criteria

A structured PICO framework was used to conduct this systematic review. The population (P) included stroke survivors at various stages of the disease, with subgroup analyses taking into consideration individuals with varying clinical characteristics (e.g., cognitive status, functional status). The intervention (I) included robot-assisted rehabilitation or exoskeleton-based therapy aimed at improving motor function of the affected upper extremity, assessed using standard clinical and instrumental tools, including: Fugl–Meyer Upper Extremity Assessment, Wolf Motor Function Test, Action Research Arm Test, Brunnstrom recovery stages of the hand, as well as muscle strength and active- and passive-range-of-motion tests. Comparison (C) included contrasts between test conditions (e.g., physiotherapy treatment with and without the use of robotics), subgroups of participants, and test repetitions to assess reliability. The results (O) focused on measurements of the functional status and motor skills of the directly affected upper limb, the reliability of repeat tests, and the accuracy of clinical assessments. This structured approach supports a comprehensive synthesis of evidence on the rehabilitation of the directly affected upper limb using robotics as a clinically relevant rehabilitation strategy for stroke survivors.

### 2.2. Search Strategy

The multi-search engine of the Main Library of the Medical University of Gdańsk was used to search for publications. The review was based on research materials obtained from six databases: PubMed, BMJ Journals, Directory of Open Access Journals, Science Direct, Research Gate, and Sage Journals. The research included the years 2019–2025. The initial search was conducted in May 2025 and repeated on August 22, 2025, before the final review. The search strategy was based on three categories of keywords: (1) terms related to the target population, (2) terms related to the area of the body receiving physical therapy treatment, and (3) terms related to rehabilitation strategies. The Boolean operator “AND” was used to combine the keyword sets. Category (1) focused on people who had a stroke and included terms such as “stroke,” “ischemic stroke,” and “hemorrhagic stroke.” Category (2) included terms related to the upper extremity directly affected, such as “upper limb,” “upper extremity,” “upper limb directly affected,” and “upper extremity directly affected.” Category (3) concerned rehabilitation strategies and included terms such as “robotic rehabilitation,” “rehabilitation using robotic,” “upper limb robotics,”. These three categories of keywords were ultimately combined in the final search using the Boolean operator “AND” to combine category (1) with category (2) and category (3). The search was limited to the title, abstract, and keyword fields.

### 2.3. Selection of Articles

Inclusion criteria are as follows:

- Only original scientific articles published in peer-reviewed scientific journals and written in English were considered.
- Eligible studies had to demonstrate a direct relationship to the topic of the review by analyzing the functionality and motor skills of the upper extremity directly affected in stroke survivors and rehabilitation strategies using robotics, with the aim of investigating the relationship between these variables.
- Included studies had to contain clearly defined results relevant to the purpose of the review.

The following studies were excluded from the review: case reports, case studies, abstracts, editorials, letters to the editor, reviews, and meta-analyses, as well as gray literature. Studies that did not analyze the relationship between the functionality and motor skills of the upper limb directly affected in stroke survivors and rehabilitation strategies using robotics were also excluded.

### 2.4. Aim and Objectives

The aim of this study is to conduct a rigorous analysis and comparison of the results evaluating the effectiveness of therapy using robots and exoskeletons to improve the motor function of the upper limb on the side directly affected in patients after a stroke, with therapy that does not use robotics. In recent years, we have seen significant advances in the technologies used in neurological rehabilitation, opening up new possibilities for individualizing the physiotherapy treatment process, more reliably monitoring rehabilitation progress, and conducting therapy in a more intensive manner focused on specific deficits faced by stroke patients [16,17]. The aim of the study is to assess the impact of robot-assisted physiotherapy on improving the motor skills and functionality of the upper limb directly affected after stroke in comparison to therapy that does not use robotics. The question asked according to the PICO model (Population, Intervention, Comparison, and Outcomes) is as follows: In patients after a stroke (P), is rehabilitation of the directly affected upper extremity using robotics (I) a more effective method of improving the functionality and motor skills of the limb (O) compared to therapy without the assistance of robots (C)?

### 2.5. Data Extractions

In order to ensure reliability, the selection of articles for the study was conducted by two independent reviewers, who identified studies that met the relevant inclusion criteria based on the abstracts of selected articles, with duplicate records being removed. Any disagreements between reviewers were resolved through discussion. If consensus could not be reached, a third reviewer was consulted. A total of 125 records were identified (PubMed—47, BMJ Journals—7, Directory of Open Access Journals—20, Science Direct—25, Research Gate—7, and Sage Journal—19) and narrowed them down to the topics listed in Section 2.1 Search strategy. Sixty-nine articles on potentially eligible studies were evaluated in detail. The following data were systematically extracted from each included study: (1) general study characteristics, including year of publication and study design; (2) participant information, such as sample size, mean age, stroke type, and disease phase; (3) details of the physiotherapy intervention for the directly affected upper limb using robotics, including duration, frequency, and whether the intervention was supervised; (4) outcome measures related to upper extremity motor function; and (5) key findings and conclusions relevant to the research question.

### 2.6. Synthesis Methods

Due to the heterogeneity of the included studies in terms of intervention protocols, outcome assessment tools, duration of therapy, and participant characteristics, it was not possible to perform a quantitative meta-analysis. Therefore, a structured narrative (qualitative) synthesis was performed to integrate the results of all included studies. The data were organized according to predefined domains, allowing for a systematic comparison of populations, interventions, and endpoints. Categorical outcomes were summarized using reported proportions and frequencies, highlighting patterns, trends, and key findings. Differences and similarities between studies were examined to provide an integrated understanding of the evidence while maintaining the integrity of the original data. All results were synthesized descriptively to support consistent interpretation and provide information for practice and future research.

## 3. Results

### 3.1. Study Selection

**Figure 1:**
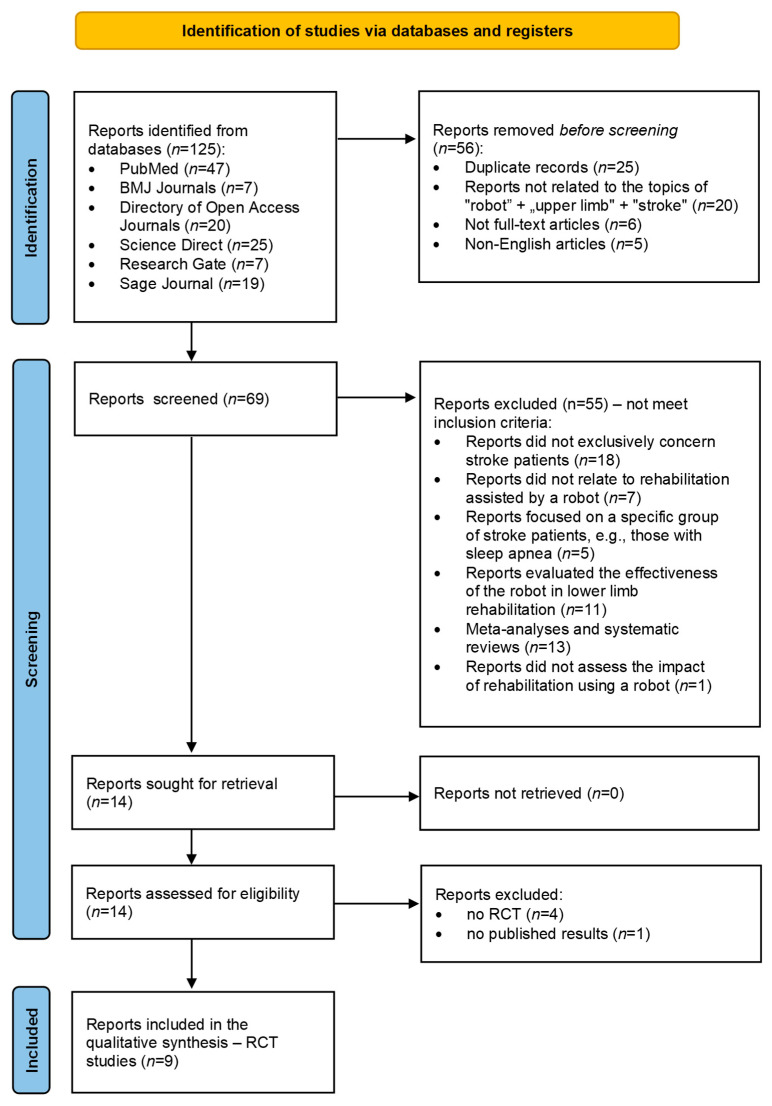
Flow chart adapted from PRISMA, which shows the process for identifying and screening the articles for inclusion and exclusion.

As a result of the search strategy used, a total of 125 potentially relevant publications were identified. After excluding duplicates (*n* = 25), publications unrelated to the topic (*n* = 20), non-English publications (*n* = 5), and publications that were not accessed in the full version (*n* = 6), 69 items remained for further analysis. After excluding studies that did not meet the inclusion criteria (*n* = 55), 14 full-text articles were evaluated. After excluding studies that were not randomized controlled trials (RCTs), 9 articles met the eligibility criteria and were included in the qualitative synthesis. The review was conducted in accordance with the PRISMA guidelines (Figure 1).

A rigorous evaluation of the studies included in the analysis shows a high degree of consistency in the criteria for inclusion and exclusion of patients in the study groups. However, differences were observed in the time elapsed since the vascular incident (Table 1).

The studies included patients in both the acute and chronic phases; in the study by Neha Singh, the age of patients ranged from 18 to 70 years [24]. Similarly, in several other studies [2,7,10,14], the age ranged from 18 to 90 and 20 to 80 years, respectively. A higher age limit was imposed for patients in the studies by L. Castelli and O. Bayindir [17,25], respectively, >55 and 35–75 years. The authors of the latest study included all patients over 18 years of age in their analysis [8].

### 3.2. Risk of Bias—RCT

A methodological analysis of the included RCTs using the PEDro scale indicates that the overall quality of the studies is moderate to good, with total scores ranging from 5 to 7 out of 10 points (Table 2). All studies met the criteria for determining inclusion criteria (point 1), randomization (point 2), similarity of groups at baseline (point 4), reporting of between-group results (point 10), and presentation of measures of central tendency and variability (point 11), indicating consistent design and reliable presentation of results. The most common methodological limitation was the lack of complete blinding of participants and therapists (points 5 and 6) in all studies, which may introduce a risk of systematic error in the measurement of intervention effects. Concealment of allocation (point 3) was not ensured in half of the trials studied, and in several studies, a full analysis according to the intention-to-treat principle (point 9) was lacking, which may also affect the reliability of the results. In addition, in some studies, less than 85% of participants originally assigned to groups were included in the analysis of selected outcomes (point 8), which limits the precision and generalizability of the results. Despite these limitations, most studies scored high in the range of 6–7 points, indicating moderately high methodological reliability and a solid basis for interpreting the effects of robotic interventions in upper limb rehabilitation after stroke. The exception is the study by Kim S. et al. (2023) [26], which scored 5 points, mainly due to deficiencies in allocation concealment, full analysis of treatment intent, and a limited number of participants with complete outcomes, suggesting greater caution in interpreting the results of this study.

In summary, the included studies demonstrate consistent and relatively robust methodology, and the intervention effects obtained can be interpreted as reliable, although certain limitations related to the lack of complete blinding and partial lack of data require moderate caution in drawing conclusions.

### 3.3. Assessment of the Quality of Evidence

An analysis of the quality assessment of the studies included in the systematic review indicates a generally moderate level of evidence quality for most of the included studies, according to the GRADE criteria (Table 3). All of the studies evaluated had some limitations in their design, mainly due to small sample sizes (*n* < 50), lack of complete blinding, and, in some cases, failure to achieve the planned sample size. These factors result in a one-level downgrade in the quality of evidence for the “risk of bias” criterion in all studies (↓1). With regard to inconsistency, all studies showed consistency of effects between groups or measurements within individual interventions, which was marked as no downgrade in quality in this aspect (↓0). Similarly, indirectness was minimal because the populations, interventions, and outcomes were directly related to the target population—stroke patients and the assessment of hand and arm function—which also did not reduce the quality of evidence (↓0). Imprecision was a significant factor contributing to the downgrading of quality in most studies. These problems were due to small sample sizes, wide confidence intervals, and limited statistical power, resulting in a one-level downgrade (↓1). No evidence of publication bias was found in any of the studies (↓0). With regard to the magnitude of effect, most studies showed clinically significant improvements in motor function, as assessed by the FMA-UE, ARAT, BBT, or G-AMA, which exceeded the minimum clinically important difference (MCID), which could increase the quality of evidence in this criterion (↑1). However, no data were found on the possible influence of confounding factors or dose–response analysis, which did not increase the quality in these criteria (↑0). Ultimately, using the GRADE method, most studies received a “moderate” rating (Moderate, ●●●○), indicating that further research may significantly affect our confidence in the effects and may change the estimated effect size. The exception is the study by Bayindur et al. (2022) [17], in which significant differences between groups were found only for some of the outcomes (BBT and pinch strength), and the short intervention time and limited samples resulted in a low-moderate quality rating (Low-Moderate, ●●○○).

In summary, despite some methodological limitations and small sample sizes, the studies provide consistent evidence of clinically significant benefits of the interventions used in hand and arm rehabilitation after stroke, but they should be interpreted with moderate caution due to the limited precision of the results.

### 3.4. Summary of Included Studies: Interventions, Measures, and Outcomes

The synthesis ultimately included nine RCTs, the descriptions of which, along with the interventions, scales, and results used, are presented below.

#### 3.4.1. Evidence of Neuroplasticity with Robotic Hand Exoskeleton for Post-Stroke Rehabilitation: A Randomized Controlled Study

In this study by Neha Singh and Nand Kumar, the effectiveness of upper limb therapy using an exoskeleton was compared with conventional therapy. The study was conducted in New Delhi between 2016 and 2019. Patients eligible for the study were between 18 and 70 years of age and had suffered a hemorrhagic or ischemic stroke between 3 and 24 months prior. Patients with any other cognitive or progressive neurological disorders were excluded from the study. The intervention was carried out over 4 weeks, five times a week for 45 min each—a total of 20 sessions. Patients were randomly assigned to a study group that trained using an exoskeleton and a control group that received conventional therapy. The effectiveness of the therapy was assessed using scales and tests such as the Modified Ashworth Scale, Active Range of Motion, Barthel Index, Brunnstrom Stage, and Fugl–Meyer Scale. In addition, neurophysiological measures of cortical excitability were examined. After both forms of therapy were used, improvement was observed in patients in both groups. However, the results in the group using the exoskeleton showed statistically greater improvement on all scales than in the control group. MAS in the RG (robot group) group changed from 1.75 ± 0.2 to 1.29 ± 0.3 and in the CG (control group) from 1.860.5 to 1.59 ± 0.6, showing a significant difference in spasticity levels between the groups—a 26% improvement in the RG and 14% in the CG. AROM also improved in both groups, but with a significant difference of 130% in the RG and 47% in the CG [24].

#### 3.4.2. Neurocognitive Robot-Assisted Rehabilitation of Hand Function: A Randomized Control Trial on Motor Recovery in Subacute Stroke

The authors of this study present research aimed at evaluating the effects of robot-assisted neurocognitive therapy and conventional therapy without the use of robots. The study took place at the Rehabilitation Center in Brissago, Switzerland, between 2013 and 2017. The therapy was conducted over 15 days spread over a period of 4 weeks, comprising 3 training sessions per day. The research group receiving robot-assisted therapy (RG) received two conventional therapy sessions and one session with a robot during the day, while the control group (CG) participated in three therapy sessions without robotic assistance. Both groups were given the same exercises, which were thoroughly explained and demonstrated by therapists. Ultimately, 33 patients aged 18–90 years were enrolled in the study, after their first and only vascular incident, which occurred no earlier than 6 weeks prior to enrollment, and with hemiplegia rated NIHS < 1. Patients presenting with aphasia, significant cognitive deficits, or upper limb pathologies such as injuries or rheumatological changes were excluded from the study. The effects of therapy were assessed using the Fugl–Meyer Scale before the start of therapy (T0), after its completion (T1), and within 8 (T2) and 32 weeks (T3) after the end of the intervention. After 4 weeks of therapy, the research group working with the robot achieved an improvement of 7.14 FMA points, while the control group achieved an improvement of 6.85 points. The data collected during the study also showed a significant improvement in further measurements in the robot-assisted group, between T0 and T2 by 7.79 FMA points, and by 8.64 FMA points between T0 and T3. The control group also showed improvement between T0 and T2 by 7.79 points and between T0 and T3 by 8.08 FMA points. At the end of the study, the RG showed an improvement of 7.14 FMA points, while the CG showed an improvement of 6.85 points [16].

#### 3.4.3. Three-Dimensional Magnetic Rehabilitation, Robot-Enhanced Hand-Motor Recovery After Subacute Stroke: A Randomized Controlled Trial

This study focuses on comparing the effects of therapy using an electromagnetic robotic system with those of traditional therapy without the use of technology. Patients eligible for the study were between 20 and 80 years of age and had experienced a vascular incident within three months or less at the time of eligibility. Patients with spasticity, significant cognitive impairment, and serious comorbidities such as pneumonia were excluded from the study. Ultimately, 40 patients were enrolled, all of whom received one hour of therapy per day for a period of one month. Half of them (RG) participated in 30 min of traditional therapy, and the remaining 30 min worked with the robot, while the control group (CG) received conventional therapy for the entire hour. In addition, both groups used other forms of therapy, such as gait re-education or strengthening exercises, for one hour per day. The Wolf Motor Function Test was used to evaluate the results. After one month of intervention in the RG, the WMFT results increased from 23.4 ± 4.1 to 34.5 ± 5.2 (*p* = 0.004), and after another month after the end of therapy, to 44.2 ± 6.6 (*p* = 0.004). In the CG, after one month, WMFT scores increased from 24.0 ± 4.5 to 30.8 ± 4.9 (*p* = 0.008), and after another month, they increased to 38.9 ± 5.7. Subsequent statistics confirmed a significant difference between the results of the individual groups (*p* = 0.018 and *p* = 0.024) [26].

#### 3.4.4. New Artificial Intelligence-Integrated Electromyography-Driven Robot Hand for Upper Extremity Rehabilitation of Patients with Stroke: A Randomized Controlled Trial

The authors present a study that aimed to investigate the effects of upper limb therapy using a robot that combines artificial intelligence technology with electromyography. The study took place in Japan between June 2020 and February 2022. Twenty patients were eligible for the study, having had their first and only vascular incident no more than 60 days prior to the date of eligibility. Additionally, the patients included in the study were between 20 and 80 years of age and were unable to move the fingers of their selectively paralyzed hand (SIAS < 2) or lift their arm on the side with paresis to nipple level (SIAS Knee Mouth Test score < 2). The intervention lasted 4 weeks, with patients in the study group participating in therapy using the above-mentioned robot twice a week for 40 min, while patients in the control group received therapy using the same robot in passive mode. The effects of therapy were assessed according to the Fugl–Meyer Assessment Motor Score (FMA). In the study group, FMA scores showed significant improvement both after 4 weeks of the study and after another 4 weeks after the end of the intervention (37.3 ± 11.2 at the beginning of the intervention, 40.6 ± 10.8 after its completion, and 41.8 ± 10.4 after another 4 weeks), while no significant difference was observed in the control group (41.1 ± 17, 43.3 ± 17.8, 45.6 ± 16.8) [27].

#### 3.4.5. Effect of Task-Oriented Training Assisted by Force Feedback Hand Rehabilitation Robot on Finger Grasping Function in Stroke Patients with Hemiplegia: A Randomized Controlled Trial

In this study, scientists investigated the effectiveness of upper limb therapy using a robot with force feedback in stroke patients with hemiplegia. Forty-four patients were enrolled in the study. The inclusion criteria for the study included a first vascular incident no more than 6 months prior to the date of enrollment, age between 20 and 80 years, and a diagnosis by a physician of central paralysis of the right upper limb/hand. The intervention lasted 4 weeks, and the exercises were performed five times a week for 40 min a day. The study group exercised with the help of the above-mentioned robot, while patients in the control group were assisted by experienced therapists. All patients were thoroughly instructed, and all tasks were explained in detail by the therapists. The therapy included, among other things, movements of grasping and transferring objects of various sizes and textures, as well as everyday tasks such as lifting a cup of water. The effects of the therapy were measured using: Fugl–Meyer Motor Function Assessment of the Hand (FMA), Action Research Arm Test (ARAT), Modified Ashworth Scale (MAS), Brunnstrom Recovery Stages of the Hand (BRSH), and Barthel Index (BI). In addition, grip strength and range of motion were also tested. After 4 weeks, the FMA hand total score, ARAT, grip strength, and range of motion of the study group improved significantly compared to the control group, with a statistically significant difference (*p* < 0.05). However, the study did not show significant differences between the groups in the MAS, BRS-H, and BI scores [11].

#### 3.4.6. Effectiveness of Upper-Limb Robotic-Assisted Therapy in the Early Rehabilitation Phase After Stroke: A Single-Blind, Randomized Controlled Study

In this article, the authors present a study aimed at determining the effectiveness of upper limb therapy in patients after a vascular incident using a robot. A total of 45 patients were ultimately enrolled in the 9-week therapy model. Patients had to meet specific inclusion criteria, such as: a first vascular incident occurring no more than one month prior to enrollment, an FMA Upper Extremity score <80%, and age over 18 years. Patients who were unable to understand instructions for working with the robot, had a stroke in the cerebellum or brain stem, or had other orthopedic or neurological conditions were excluded from the study. The control group underwent conventional therapy focused on motor skills, which was adapted to their needs and abilities. The study group also participated in conventional therapy, but 25% of all sessions during the week were replaced with exercises using the robot. In total, the study group took part in 36 sessions with robotic assistance. The patients in the study group took part in exercises that were game-based and required them to move the affected limb along a specific trajectory, passing through designated points, with the assistance of the robot. The assessment took place before the start of therapy (T0), after its completion (T1), and 6 months after the last training session (T2) using the FMA-UE, Box and Block Test (BBT), and Wolf Motor Function Test (S-WMFT). After 9 weeks of therapy, the FMA-UE scores in the study group improved from 32.4 (T0) to 51.9 (T1) and 57.1 (T2), while in the control group, improvement was only observed between T0 and T1, with scores increasing from 31.6 to 42.4. Both groups also showed improvement in BBT by 14.3% (T1) and 17.3% (T2) in the study group and by 11.7% between T0 and T1 in the control group. S-WMFT between T0 and T1 showed an improvement of 30.1% in the study group and 33.1% in the control group [13].

#### 3.4.7. The Effect of Adding Robot-Assisted Hand Rehabilitation to Conventional Rehabilitation Program Following Stroke: A Randomized Controlled Study

The authors present a study aimed at determining the effectiveness and improvement of rehabilitation outcomes in the upper limb directly affected in patients after stroke, following the introduction of elements of robot-assisted therapy compared to traditional therapy methods. The study took place at Marmara University in Turkey in 2012, and 33 patients were enrolled, who were assigned to the study group and the control group. To be included in the study, patients had to be between 35 and 75 years of age, obtain a Fugl–Meyer Assessment score of 30–56, a Modified Ashworth Scale score of <2, and have had their first cerebrovascular accident less than 3 months prior to the date of qualification. The intervention lasted 5 weeks, during which both groups participated in conventional rehabilitation sessions twice a week, and the study group additionally underwent one hour of robot-assisted therapy. During the one-hour session with the robot, patients performed exercises such as passive/active or assisted exercises to increase range of motion, muscle strengthening exercises, and exercises for everyday functions. Progress in therapy was assessed using the Fugl–Meyer Assessment, Box and Block Test, and hand grip strength, while patients were examined at the beginning of the intervention, immediately after its completion, and three months after the end of the study. Both groups made significant progress, but in the research group, results in tests such as BBT and pinch strength parameters were significantly better after a 5-week intervention than in the control group (*p* < 0.05). In addition, all results (except FMA) in the study group showed a statistically greater improvement 3 months after the study than in the control group [17].

#### 3.4.8. The Impact of Robotic Hand Rehabilitation on Hand Function and Fatigue in Patients with Stroke

The study focused on assessing the potential and impact of using a robot in the therapy of the upper limb directly affected in stroke patients. Twenty-four patients who had suffered their first vascular incident between 6 and 12 months prior to the date of qualification and were over 55 years of age were eligible for the study. Patients who were unable to understand and follow the therapist’s instructions, had cognitive impairments, scored 19 on the Motoricity Index Upper Limb, and scored 4 or higher on the Modified Ashworth Scale were excluded from the study. The intervention lasted 4 weeks, with the control group performing exercises aimed at restoring upper limb functionality three times a week for 45 min. The study group exercised using a robot at the same frequency with additional elements of conventional therapy. During robot-assisted therapy, patients performed exercises such as passive mobilization, active mobilization, interactive games, and exercises focused on memory and coordination. Motor progress was assessed using the Motor Impairment—Upper Limb (MI-UL), Fugl–Meyer Assessment (FMA-UE), and Stroke Upper Limb Capacity Scale (SULCS), and patients were examined before the start of the study and after the completion of the 4-week intervention. The study showed that both groups experienced statistically significant improvement, but a more detailed comparison of the results of both groups revealed that the study group achieved greater progress in terms of MI-UL (*p* < 0.001), FMA-UE (*p* < 0.001), and SULCS (*p* < 0.001) [25].

#### 3.4.9. Effects of Robot-Assisted Rehabilitation on Hand Function of People with Stroke: A Randomized, Crossover-Controlled, Assessor-Blinded Study

The aim of this study was to evaluate the effects of rehabilitation in stroke patients using robotic therapy methods and to compare them with the results of patients exercising without the use of a robot. Twenty-two patients who had suffered their first vascular incident at least three months prior to the date of qualification, aged between 20 and 75, were eligible for the study. Patients had to understand commands, have sensory disorders, present hemiplegia, and have muscle tone enabling movement (Modified Ashworth Scale < 3). Patients who were unable to see or hear the device’s feedback accurately and those with comorbidities that could directly affect motor skills and mobility were excluded from the study. The intervention lasted 6 weeks, and each group performed ADL-focused exercises in 60 min training sessions twice a week. Both the control and study groups performed similar exercises, such as passive movements of individual parts of the hand and those focused on specific functions and spatial movements, assisted by a robot in the study group and only a therapist in the control group. Progress was assessed using the Fugl–Meyer Assessment Scale (FMA-UE), Box and Block Test (BBT), and Modified Barthel Index (MBI). Both groups showed improvement, with the greatest improvement in MBI, where the study group achieved significantly better results after the intervention than the control group (*p* = 0.038). In terms of FMA-UE, a significantly greater improvement was observed in the study group (proximal *p* = 0.30 and total *p* = 0.46). No significant differences between the groups were observed in terms of BBT [14].

A comprehensive comparison of the results of the individual studies mentioned above is presented in Table 4.

An analysis of nine studies evaluating upper limb rehabilitation after stroke demonstrated that interventions led to improved motor function in both groups, with the experimental group (EG) often achieving greater improvements. In Singh (2021), the EG achieved a significant increase in FMA-UE (36 → 50.2; *p* = 0.0004) and a reduction in MAS spasticity (1.75 → 1.29), while the CG also improved, but to a lesser extent (FMA-UE 37.4 → 45.4; *p* = 0.0009) [24]. In Ranzani (2020), a significant increase in FMA-UE was observed in both groups (↑7–8 points), but the EG’s advantage was only in FMA-UE, while no significant difference between the groups was found in BBT [16].

In Kim’s study (2023), the EG achieved a significant improvement in WMFT manual function tests (23.4 → 34.5; *p* = 0.004) and FMA-UE (28.5 → 39; *p* = 0.024 between groups), indicating the superiority of the EG over the CG [26]. Murakami (2023) showed a small, insignificant increase in FMA-UE in the EG and no differences between groups [27]. In Dehem (2019), the EG outperformed the CG in the BBT and S-WMFT manual tests, although the differences in FMA-UE were insignificant [13].

Studies by Castelli (2025) and Li (2024) showed a significant advantage of the EG in FMA-UE (*p* = 0.002; *p* < 0.05), MI-UL (*p* < 0.001), and ARAT and ROM, with a simultaneous reduction in MAS spasticity in both groups (Castelli 2025) [11,25]. In the study by Bayındır (2022), the improvement in manual function, precision, and grip strength was greater in the EG, as in Lee (2021), where the EG (hand therapy) achieved better results in MBI (*p* = 0.038), although FMA-UE showed no significant difference between the groups [14,17].

In summary, rehabilitation interventions led to significant improvements in upper limb function, with EG showing particular advantages in terms of manual function, strength, and precision. The effects on spasticity were less consistent, requiring further research to determine the optimal type and intensity of therapy. It should be noted that the analysis includes studies with different intervention protocols and participant characteristics. For example, Singh (2021) and Ranzani (2020) differed in both the post-stroke phase of the patients (from 3 weeks to more than 6 weeks) and the duration and frequency of the sessions, making it difficult to directly compare the effects [16,24]. The variety of assessment tools used—from FMA-UE and MAS, through WMFT and ARAT, to MI-UL and fine motor skill tests (BBT, NHPT, and JTHFT)—limits the possibility of a synthetic assessment of the effectiveness of interventions in a single functional dimension.

Table 5 summarizes publications based on randomized controlled trials (RCTs) and their characteristics according to the PRISMA methodology.

In summary, all studies have shown that both traditional physical therapy and robot/exoskeleton therapy lead to improved upper limb function after stroke. In most studies (Singh 2021, Kim 2023, Castelli 2025, Bayındır 2022, Li 2024), the use of robots was associated with greater improvements in motor function, reduced spasticity, or improved grip strength and precision compared to the control group [11,17,24,25,26]. However, the results were not consistent, and in some studies (Murakami 2023, Dehem 2019, Lee 2021), the differences between the groups were not statistically significant, which may be due to the short duration of the intervention, small group sizes, or differences in treatment protocols [13,14,27].

## 4. Discussion

The systematic review assesses the motor and functional effects of rehabilitation using robots and exoskeletons on the upper limb directly affected by neurological syndrome in patients after stroke. An analysis of nine studies on upper limb rehabilitation after stroke indicates that therapeutic interventions lead to significant improvement in motor function in both groups, with experimental groups (EGs) often achieving greater benefits, especially in terms of manual function, strength, and grip precision. This is indicated, in particular, by the results of Singh (2021), Ranzani (2020), Kim (2023), Castelli (2025), Li (2024), and Bayındır (2022) [11,16,17,24,25]. The effects of interventions on spasticity were less consistent, with Murakami (2023) and Lee (2021) showing no clear advantage for EGs in FMA-UE [14,27]. Studies have shown that, in addition to pharmacotherapy, it is physiotherapy that improves the functionality of directly affected limbs, counteracts muscle atrophy and joint stiffness, but most importantly, improves the quality of life of patients after a stroke [28]. That is why it is so crucial to constantly search for modern methods of working with patients that will enable them to regain as much functionality and independence as possible in their lives after leaving the medical center [29]. The use of robots is precisely such a progressive approach to working with stroke patients, allowing us to enforce the repetitiveness/intensification of movements and thus introduce repetitiveness into therapy, which brings very good results in the context of rebuilding neural networks and limb function. In combination with function-based work and specific motor tasks, appropriately supported by robots, in the form of active patient work, we are able to achieve lasting changes in the neural networks of the motor system, improve correct limb patterns, and rebuild function [8,11].

Research shows that there is a phenomenon of increased neuroplasticity immediately after a vascular incident, during which rapid rehabilitation can bring significantly better results [8,13,30]. In the case of upper limb rehabilitation, the first two weeks after the incident seem to be crucial for achieving the best results [31]. In the studies included in the review, the time interval between the vascular incident and the study ranged from 4 to 6 weeks to as long as 24 months. However, most of the studies were conducted on patients in the subacute phase, which highlights the need for further studies including patients in the chronic phase and in more homogeneous groups [11,13,14,16,17,26,27]. In addition, the patients included in the studies had different abilities and motor skills on the day of inclusion. In most studies, the key criteria for patient inclusion were the ability to understand commands, a score of 1/1+/2 on the Ashworth Scale, or the ability to maintain a sitting position independently. However, the authors of some studies initially required qualified patients to have specific skills, e.g., <80% on the Fugl–Meyer Scale or the ability to move individual fingers within specific ranges of motion [11,13]. Differences in the criteria for the motor abilities of patients qualified for the studies, therefore, greatly differentiate the results of the studies. It therefore seems necessary to standardize certain protocols concerning both patient qualification and the course of rehabilitation itself, so that the results are as reliable as possible.

In some respects, rehabilitation using robots achieves results comparable to those achieved using conventional methods. In a study by Yinghua Li, the motor skills of both groups were tested using a number of different scales [11]. A noticeable improvement in the group receiving robot-assisted therapy was observed in terms of FMA hand total score, ARAT, grip strength, and active range of motion. No significant differences were observed in the case of BRS-H, the Modified Ashworth Scale, and the Barthel Index. It is noteworthy that this particular study focused on hand grip strength and used a robot that covered the upper limb below the elbow. The study described by Ozun Bayindir also used a robot model covering only the hand and wrist, where the study focused on assessing the improvement of the entire limb function [17]. Scales such as the FMA, Jebsen–Taylor Hand Function Test, and Box and Block Test were used, and a statistically significant advantage of the experimental group was observed only in the case of scales and tests covering finger and wrist functions. A completely different set of results is presented in a study by Neha Singh, in which the research group exercising with the use of a robot showed substantially greater statistically significant results of therapy [24]. The study used scales such as the Fugl–Meyer Scale and the Modified Ashworth Scale, and also examined the active range of motion and cortical excitability in the ipsilesional hemisphere—in all of the above, it was the study group that achieved better results. All studies differed in terms of exercise protocols, the initial condition of the patients included in the study, the size of the groups, and the robots used, which explains the differences in the results obtained and implies the need for further research.

Many rehabilitation protocols included in the analysis incorporated task-oriented exercises, which are considered extremely effective in improving upper limb motor function in stroke patients. Functional tasks help to rebuild the reflex loops of the central nervous system, and through a functional approach to a specific motor task, they facilitate the performance of ADLs, which enable patients to adapt to changing conditions around them, increase their independence, and additionally act as a motivating factor [11]. The review included studies in which exercise protocols were based mainly on the use of task-oriented exercises. They focused mainly on the use of cylindrical and spherical grips, and the tasks included activities such as transferring a ball to a bucket, lifting a cup to the mouth, and moving cylindrical pegs to the appropriate places on a board. In a study by O. Bayindir, the research group used a robot to participate in computerized goal-oriented games, such as following a target or imitating a basketball game, as an addition to conventional therapy elements, including stretching, exercises focused on active and passive range of motion, and strengthening exercises [17].

The review also includes studies that lacked the element of purposeful exercise and connection with ADLs [16,24,26,27]. The study protocol included exercises for the range of motion of the affected limb, finger stretching, sensory stimulation, and strengthening exercises for the control group. With the assistance of the robot, patients in the study group performed, among other things, finger flexion/extension and thumb opposition movements [26]. In addition, proprioception was stimulated using magnetic forces, and counter movements were performed using the thumb and the other four fingers of the affected limb to stimulate functional grip within the hand [26]. All exercises using the robot were based on the active assistance of the patient in performing the movement independently, and only when more support was needed did the robot perform or complete the movement. The study focused on enforcing appropriate movements within the fingers and hand using technology that enabled continuous analysis of finger position and the performance of assisted movements adapted to the patient and their capabilities. As a result, it was the group training with the robot that achieved better motor and functional outcomes compared to the control group. Even a month after the end of the intervention, patients continued to regain normal hand function and achieved better long-term results in terms of both function and independence in performing various everyday tasks [26].

Rehabilitation using robots allows for the inclusion of constant and highly repeatable support for specific patterns in therapy, compared to conventional therapy. Feedback received by the patient is important in this type of rehabilitation. Robots that only passively guide the patient’s limb provide visual feedback on the shape or weight of the object being lifted during the exercise. Force feedback robots provide patients with additional feedback on force or direction and assist in performing a specific movement. It has been proven that this type of work is effective and brings additional results compared to conventional work [11]. In a study by Neha Singh, a robot was used to assist the patient’s movements, and it was the study group that achieved statistically greater improvement in functional tests than the control group [24]. In the case of devices that not only assist movement but also provide force feedback in terms of strength and grip ability, the differences between the groups were not as obvious. It is therefore necessary to increase the amount of research and possibly use continuous technological progress to construct devices that both guarantee a wider range of feedback for patients and cover the entire limb affected by the neurological syndrome.

The most recent meta-analysis, encompassing 54 randomized clinical trials (n = 2744), showed that robotic upper limb rehabilitation leads to a small but statistically significant improvement in motor function compared to conventional therapy (SMD = 0.14); however, this effect does not reach clinical significance and is not sustained in long-term follow-up [22]. Importantly, the same analysis found no significant differences between robotic and conventional therapy in terms of activities of daily living (ADLs), both immediately after the intervention and at follow-up. Similar conclusions have been reported in other recent meta-analyses. A review by Yang et al. (2023) showed that robot-assisted therapy leads to improved upper limb motor function; however, the magnitude of the effect depends on the intensity and duration of therapy, suggesting that the benefits may stem more from an increased “dose” of rehabilitation than from the specific nature of the technology itself [32]. In contrast, a network analysis by Wang et al. (2024/2025) indicates that the effectiveness of robots varies depending on the phase of stroke, with greater effects observed in the acute and subacute periods than in the chronic phase [33]. These results are consistent with the observations of Wang et al. (2026), who demonstrated that the response to robotic therapy is strongly dependent on the stage of the recovery process and the degree of preserved motor function [19].

In terms of how robots work, recent meta-analyses highlight their positive impact on “body functions and structures,” particularly in terms of muscle strength and motor control [22]. At the same time, these effects do not translate proportionally into levels of activity and participation, which remains one of the main limitations of this form of therapy. When comparing robotic rehabilitation with other modern technologies, such as virtual reality, it should be noted that some meta-analyses indicate a more pronounced effect of VR on motor function, spasticity, and quality of life [8]. This may suggest that the motivational and cognitive components present in immersive therapies play a significant role in the rehabilitation process and may serve as an important complement to robotic therapy.

Robotic rehabilitation is a modern approach to upper limb rehabilitation and shows truly promising results. Further research on larger groups of patients, with more homogeneous groups and standardized rehabilitation protocols, is still necessary in order to accurately assess its effectiveness compared to conventional therapy. With the growing number of stroke survivors, both clinicians and physiotherapists agree on the need for continuous development of new techniques and forms of rehabilitation that could reduce disability rates among stroke patients. Rehabilitation using robots is one such modern solution that has been proven to be effective by scientific research due to the significant intensification of movement, high repeatability of movement sequences, and the implementation of functional tasks. However, this technology is not widely available, mainly due to the high cost of the robots themselves and the infrastructure they require. In Wagner’s study (2011), the average cost of delivering robot therapy and intensive comparison therapy is $5152 and $7382, respectively (*p* < 0.001), and both are significantly more expensive than usual care alone (no additional intervention costs) [34]. This includes both the space they require and the appropriate technology, such as computers or even constant access to the Internet. In addition to these costs, it is also necessary to have physiotherapists who are properly trained to operate such technologies and who can effectively utilize both the full potential of the device and the patient. All these factors significantly hinder the development of larger-scale research, the introduction of this technology into more institutions, and an increase in the number of patients who could benefit from these technologies [24].

## 5. Conclusions

Upper extremity rehabilitation after stroke using robots and exoskeletons shows promising potential for improving motor function, particularly in terms of manual dexterity, strength, and grip precision. In many of the analyzed studies, greater benefits were observed in groups receiving robot-assisted therapy compared to conventional therapy, although the results were not consistent across all assessed areas, particularly regarding spasticity.

At the same time, the significant heterogeneity of the studies—including differences in patient characteristics, post-stroke phase, therapeutic protocols, and assessment tools used—limits the ability to draw definitive conclusions. Additionally, small sample sizes and the lack of long-term follow-up limit the generalizability of the results.

In summary, robotic-assisted rehabilitation may serve as a valuable supplement to standard therapy methods; however, its actual efficacy and optimal application require further large-scale studies with standardized protocols and longer follow-up periods.

## 6. Limitations

As highlighted earlier, this systematic review has certain limitations. The studies were characterized by heterogeneity of participants, both in terms of age and the post-stroke phase. There were also differences in the duration of the intervention and the type of therapy used, including robotics, manual training, and various exercise protocols. Such diversity makes it difficult to directly compare results between studies.

In addition, individual studies used a variety of tools to assess upper limb function, including manual tests (WMFT, BBT, S-WMFT, NHPT, and JTHFT), spasticity assessment scales (MAS), and measurements of strength and range of motion (ROM, grip strength, and pinch). The lack of standardization of tools limits the possibility of unambiguous comparison of intervention effects between groups and between studies.

Another limitation is the small number of participants in most studies (20 to 45 people), which affects the statistical power of the analyses and limits the generalizability of the results to a wider population of stroke patients.

The interventions in the analyzed studies were conducted over a relatively short period of time—from 4 to 9 weeks—and the long-term effects of therapy were not monitored in most cases. This limits the ability to assess the durability of the improvement in upper limb function.

In some studies, the reporting of results was incomplete—not all studies presented complete data on spasticity, muscle strength, or manual test results, which made it difficult to fully assess the intergroup effects.

Finally, the lack of standardization of therapeutic protocols, in terms of intensity, frequency, and type of exercises performed, limits the ability to draw general conclusions about the effectiveness of interventions.

In light of the above limitations, it is necessary to conduct larger, multicenter randomized trials involving standardized treatment protocols and assessment tools, with long-term monitoring of outcomes. These studies should take into account the different phases after stroke and the diversity of patients in order to determine the optimal parameters of intervention and their impact on upper limb function. This approach will allow for more precise, reliable, and generalizable conclusions regarding the effectiveness of post-stroke rehabilitation.

## Figures and Tables

**Table 1:** Inclusion and exclusion criteria for patients enrolled in the study programs.

Inclusion criteria:
1. Age: 18–70 [1], 18–90 [2], 20–80 [3,4,5], >18 [6], 35–75 [7], >55 [8].
2. First brain vascular incident confirmed by imaging
3. Modified Ashworth Scale score < 2
Exclusion criteria:
1. Cognitive impairment
2. Aphasia
3. Orthopedic injuries, rheumatic diseases
4. Inability to understand and follow simple instructions from the therapist
5. Uncontrolled hypertension
6. Depression
7. Stroke in the brain stem or cerebellum
8. Neglect
9. Inability to maintain an independent sitting position

**Table 2:** Assessment of systematic error risk using the PEDro scale.

No.	PEDro Scale	Singh N. et al., 2021 [24]	Ranzani R. et al., 2020 [16]	Kim S. et al., 2023 [26]	Murakami Y. et al., 2023 [27]	Dehem S. et al., 2019 [13]	Castelli L. et al., 2025 [25]	Bayindur O. et al., 2022 [17]	Li Y. et al., 2024 [11]	Lee H.-C. et al., 2021 [14]
1	Eligibility criteria were specified	yes	yes	yes	yes	yes	yes	yes	yes	yes
2	Subjects were randomly allocated to groups (in a crossover study, subjects were randomly allocated an order in which treatments were received)	yes	yes	yes	yes	yes	yes	yes	yes	yes
3	Allocation was concealed	yes	no	no	no	yes	yes	yes	no	no
4	The groups were similar at baseline regarding the most important prognostic indicators	yes	yes	yes	yes	yes	yes	yes	yes	yes
5	There was blinding of all subjects	no	no	no	no	no	no	no	no	no
6	There was a blinding of all therapists who administered the therapy	no	no	no	no	no	no	no	no	no
7	There was a blinding of all assessors who measured at least one key outcome	yes	yes	yes	yes	yes	yes	yes	yes	yes
8	Measures of at least one key outcome were obtained from more than 85% of the subjects initially allocated to groups	yes	no	no	yes	no	yes	yes	yes	yes
9	All subjects for whom outcome measures were available received the treatment or control condition as allocated or, where this was not the case, data for at least one key outcome were analyzed by “intention to treat”	no	yes	no	yes	yes	no	no	no	yes
10	The results of between-group statistical comparisons are reported for at least one key outcome	yes	yes	yes	yes	yes	yes	yes	yes	yes
11	The study provides both point measures and measures of variability for at least one	yes	yes	yes	yes	yes	yes	yes	yes	yes
Sum score:		7	6	5	7	7	7	7	6	7

**Table 3:** Assessment of the quality of evidence according to GRADE guidelines—RCT.

Article	Risk of Bias (Limitations in Study Design/Execution)	Inconsistency	Indirectness	Imprecision	Publication Bias	Large Magnitude of Effect	Confounding	Dose–Response	GRADE Quality	Justification
Singh N. et al., 2021 [24]	↓1—Randomization present, no full blinding, small sample (*n* = 27)	↓0—Results consistent across groups	↓0—Population and intervention directly relevant	↓1—Small sample, wide CIs	↓0—No evidence	↑1—FMA-UE improvement above MCID	↑0—No data on confounding	↑0—No dose–response assessment	●●●○ Moderate	Well-designed RCT; small sample and lack of blinding reduce certainty; clinically important effect increases confidence
Kim S. et al., 2023 [26]	↓1—Randomization, no full blinding, some drop-outs, small sample (*n* = 36)	↓0—Results consistent	↓0—Direct population and intervention	↓1—Sample *n* = 32, some wide CIs	↓0—No evidence	↑1—FMA-UE change ≥ MCID	↑0—No confounding data	↑0—No dose–response	●●●○ Moderate	RCT conducted properly but small sample; clinically meaningful effects, no publication bias
Ranzani R. et al., 2020 [16]	↓1—Randomization, no full blinding, target sample not reached	↓0—Results consistent	↓0—Direct population	↓1—Small sample (*n* = 20 instead of planned 30), wide CIs	↓0—No data	↑1—Clinically significant improvements in FMA and MAS	↑0—No confounding data	↑0—No dose–response	●●●○ Moderate	Limited by small sample; clinically meaningful effect, no evidence of confounding
Murakami Y. et al., 2023 [27]	↓1—Randomization, single-blinded, small sample (*n* = 20), limited power	↓0—Results consistent	↓0—Direct population (post-stroke)	↓1—*n* < 30, some marginal outcomes	↓0—No evidence	↑1—FMA improvement clinically relevant	↑0—No data	↑0—No dose–response	●●●○ Moderate	Well-designed RCT, small sample; clinically important improvements, no publication bias
Li Y. et al., 2024 [11]	↓1—Randomization, single-blinded, small sample (*n* = 44), some drop-outs	↓0—Results consistent	↓0—Direct population and intervention	↓1—Sample size small for subgroup analysis	↓0—No evidence	↑1—FMA-Hand, ARAT, grip strength improvements clinically relevant	↑0—No confounding data	↑0—No dose–response	●●●○ Moderate	RCT with consistent outcomes; small sample reduces certainty; effect sizes clinically relevant
Dehem S. et al., 2019 [13]	↓1—Randomization, single-blinded, moderate drop-outs, pragmatic trial	↓0—Results consistent	↓0—Population directly relevant	↓1—Sample *n* = 32 at post-intervention, 28 at follow-up	↓0—No evidence	↑1—Large effect in social participation (Cohen’s d = 0.88), moderate in motor ability	↑0—No data	↑0—No dose–response	●●●○ Moderate	Well-conducted RCT; moderate drop-out and small sample reduce certainty; effect size substantial for social participation
Castelli L. et al., 2025 [25]	↓1—Single-blind, small sample (*n* = 24), pilot study; randomization applied; some differences in conventional treatment duration	↓0—Results consistent across outcomes	↓0—Population and interventions directly relevant to post-stroke hand rehab	↓1—Small sample, wide SDs for some measures	↓0—No evidence of selective publication	↑1—G-AMA achieved clinically meaningful improvements in motor performance, autonomy, and fatigue compared to G-CON	↑0—No evidence of confounding analysis	↑0—No dose–response assessment	●●●○ Moderate	Pilot RCT with small sample; consistent improvements in multiple domains (FMA-UE, MI-UL, SULCS, mBI, and MFIS) increase confidence; lack of full blinding and small sample lower certainty
Bayindur O. et al., 2022 [17]	↓1—Single-blind, small sample (*n* = 33), short intervention (5 weeks), same therapist for both groups; randomization applied	↓0—Results consistent across outcomes	↓0—Population (post-stroke patients with mild-to-moderate UL deficits) and intervention (robot-assisted hand therapy) directly relevant	↓1—Small sample, some outcomes with variable results (wide IQR), short follow-up	↓0—No evidence of selective publication	↑0—only BBT and pinch strength showed significant differences; other outcomes (FMA, 9HPT, JHFT, grip strength) showed no significant intergroup differences	↑0—No evidence of confounding analysis	↑0—No dose–response assessment	●●○○ Low-Moderate	Small RCT; robot-assisted therapy improved BBT and pinch strength compared to control, but most outcomes showed no significant differences; short intervention duration and small sample size reduce confidence in effect estimates
Lee H.-C. et al., 2021 [14]	↓1—Randomization present, pilot study, small sample (*n* = 24), not fully double-blinded	↓0—Results consistent across all outcomes	↓0—Population and intervention directly relevant	↓1—Small sample, wide SDs for some measures	↓0—No evidence of selective publication	↑1—Clinically meaningful improvements across multiple outcomes (FMA-UE, mBI, MFIS)	↑0—No data on confounding factors	↑0—No dose–response assessment	●●●○ Moderate	Pilot RCT; small sample limits certainty; lack of full blinding lowers confidence; however, clinically meaningful improvements in multiple domains increase the certainty of evidence by one level

**Table 4:** Comparative summary of the results of individual studies included in the review. Abbreviations: EG—experimental Group, CG—control group, FMA-UE—Fugl–Meyer Assessment for Upper Extremity, MAS—Modified Ashworth Scale, AROM—Active Range of Motion, MI-UL—Motor Imagery for Upper Limb, BBT—Box and Block Test, WMFT—Wolf Motor Function Test, MBI—Motor Activity Log/Modified Barthel Index, NHPT—Nine-Hole Peg Test, JTHFT—Jebsen–Taylor Hand Function Test, ROM—Range of Motion, S-WMFT—Simplified Wolf Motor Function Test, EMG—electromyography.

Author (Year)	Assessment Tools	FMA-UE (Change; Significance)	Manual Function Tests	Spasticity (MAS)	Strength/ROM	Between-Group Differences
Singh 2021 [24]	FMA-UE, MAS, AROM, Brunnstrom	EG: 36 ± 7.7 → 50.2 ± 6.5 (*p* = 0.0004); CG: 37.4 ± 9.1 → 45.4 ± 9.7 (*p* = 0.0009)	—	EG: 1.75 ± 0.2 → 1.29 ± 0.3 (↓)	AROM ↑ in both groups	Greater improvement in EG (FMA-UE, MAS)
Ranzani 2020 [16]	FMA-UE, BBT, MAS	↑ 7–8 points in both groups (significant)	BBT: +11.43 (EG) vs. +12.85 (CG) blocks/min	EG: +0.07; CG: −1.54	—	Significant difference in FMA-UE; no EG advantage in BBT
Kim 2023 [26]	WMFT, FMA-UE, MBI	EG: 28.5 ± 4.8 → 39 ± 5.6; CG: 29.2 ± 5.1 → 35.8 ± 6.0 (*p* = 0.024 between groups)	WMFT: EG 23.4 ± 4.1 → 34.5 ± 5.2; CG 24.0 ± 4.5 → 30.8 ± 4.9	—	—	Significant advantage of EG (WMFT *p* = 0.018; FMA-UE *p* = 0.024)
Murakami 2023 [27]	FMA-UE, MAS	Non-significant increase in EG; no significant change in CG	—	No significant changes	—	No clear between-group differences
Dehem 2019 [13]	FMA-UE, BBT, S-WMFT	No between-group differences in FMA-UE	BBT: greater improvement in EG; S-WMFT: greater improvement in EG	—	—	EG advantage in BBT and S-WMFT
Castelli 2025 [25]	FMA-UE, MAS, MI-UL	Significant increase in both groups; greater in EG (*p* = 0.002)	—	MAS decreased in both (EG *p* = 0.009; CG *p* = 0.024)	MI-UL: greater increase in EG (*p* < 0.001)	Significant EG advantage (FMA-UE, MI-UL, MAS)
Bayındır 2022 [17]	FMA-UE, BBT, NHPT, JTHFT, grip strength, pinch	Improvement in both groups	BBT: EG advantage (*p* < 0.05); NHPT and JTHFT improved	—	Grip strength ↑; pinch: EG advantage (*p* < 0.05)	EG advantage in precision and strength
Li 2024 [11]	FMA-UE, ARAT, ROM, grip strength, MAS	Significant improvement in both; EG superior (*p* < 0.05)	ARAT: significant improvement; EG superior	No significant changes	ROM and strength ↑ in both	Significant EG advantage (FMA-UE, ARAT, ROM, strength)
Lee 2021 [14]	FMA-UE, BBT, EMG, MBI	Improvement in both (*p* = 0.46 between groups)	BBT: improvement; no clear robot advantage	—	EMG: improved activation	Better results in therapy group (MBI *p* = 0.038)

**Table 5:** Summary of nine randomized controlled trials comparing rehabilitation of the upper limb directly affected by neurological syndromes using a robot and without its use.

Authors/Year	Participants	Intervention	Outcomes Measurment	Results
Singh N. et al., 2021 [24]	*N* = 23 (19 men, 4 women) *N* = 12 (in experimental group) Mean age (SD) of 41.1 years Adults with ischemic or hemorrhagic stroke within 3–24 months prior.	Both the control and study groups participated in physiotherapy treatment spread over 20 sessions lasting 45 min over a period of 4 weeks. The study group exercised using an exoskeleton that assisted with extension and flexion movements of the wrist and fingers.	FMA-UE (Fugl–Meyer Assesment Upper Extremity) Brunnstrom Stage Scale MAS (Modified Ashworth Scale) AROM (Active Range of Motion)	Both groups showed significant improvement compared to baseline values, but greater differences were observed in the study group. MAS in GB changed from 1.75 ± 0.2 to 1.29 ± 0.3, showing significantly less spasticity in patients in this group. AROM increased significantly in both groups, from 150 ± 9.70 to 34.60 ± 14.50 in the RG (*p* = 0.0004) and from 13.6 ± 7.70 to 20.00 ± 8.10 in the CG (*p* = 0.002).
Ranzani R. et al., 2020 [16]	*N* = 27 (18 men, 9 women) *N* = 14 (in experimental group) Mean age (SD) of 70 in experimental group and 67.46 in control group Adults with ischemic or hemorrhagic stroke more/further than 6 weeks prior.	Both groups participated in physiotherapy treatment during 15 training sessions spread over a period of 4 weeks. On each of these days, they took part in three exercise sessions (two 45 min sessions and one 30 min session). Both groups received a similar set of exercises, but one training session in the study group was conducted with the use of a robot.	FMA-UE (Fugle-Meyer Assessment of the Upper Limb) BBT (Box and Block Test) MAS (Modified Ashworth Scale)	The FMA-EU scores increased in both the research/control groups by 7.14/6.85, 7.79/7.31, and 8.64/8.08 points, making these differences between the groups statistically significant. BBT increased by an average of 11.43 blocks/minute in the study group and 12.85 blocks/minute in the control group. MAS increased by 0.07 points in the study group and decreased by 1.54 points in the control group.
Kim S. et al., 2023 [26]	*N* = 36 (17 men, 19 women) *N* = 18 (in experimental group) Mean age (SD) of 60.5 in experimental group and 61.3 in the control group) Adults with ischemic or hemorrhagic stroke less than 3 months prior.	Both groups participated in physiotherapy sessions every day for a month, for 30 min a day. The study group exercised using a robot under the supervision of an experienced therapist, while the control group participated in occupational therapy sessions aimed at restoring upper limb motor function.	WMFT (Wolf Motor Function Test) FMA-UE (Fugl–Meyer Assesment of upper limb) MBI (Modified Barthel Index)	In the study group, WMFT scores increased from 23.4 ± 4.1 to 34.5 ± 5.2 after one month of therapy and increased significantly at follow-up (*p* = 0.004). In the control group, they increased from 24.0 ± 4.5 to 30.8 ± 4.9 and also significantly at follow-up (*p* = 0.010); the results between the groups were statistically significant (*p* = 0.018). FMA-UE in the study group increased from 28.5 4.8 to 39 5.6 after a month of intervention, while in the control group an increase from 29.2 5.1 to 35.8 6.0 can be observed. Significant differences between the groups were observed (*p* = 0.024).
Murakami Y. et al., 2023 [27]	*N* = 20 (14 men, 6 women) *N* = 11 (in experimental group) Mean age (SD) of 50.4 in experimental group and 49.8 in the control group. Adults with ischemic or hemorrhagic stroke more than 60 days prior.	Both groups exercised with the robot twice a week for four weeks, with each training session lasting 40 min. The research group exercised actively with the robot, i.e., the robot assisted the patient’s movements. In the control group, the robot passively performed movements with the patient’s limb without the need for patient involvement.	FMA-UE (Fugl–Meyer Assesment of Upper Limb) MAS (Modified Ashworth Scale).	Within the research group, a significant increase in FMA-EU (*p* = 0.11 and 0.21, respectively) can be observed, while no statistically significant differences were noted in the control group.
Dehem S. et al., 2019 [13]	*N* = 45 (21 men, 24 women) *N* = 23 (in experimental group) Mean age (SD) 67.3 in experimental group and 68.6 in control group. Adults with ischemic stroke up to one month prior.	Both groups participated in a 9-week intervention. The control group received therapy adapted to their needs without the use of a robot, while in the experimental group, 25% of the weekly training sessions were performed with the help of a robot. The exercises included limb movements along appropriately designated trajectories, assisted by the robot.	FMA-UE (Fugl–Meyer Assessment of the Upper Limb) BBT (Box and Block Test) S-WMFT (Wolf Motor Function Test)	BBT showed a significantly greater improvement in the study group from 3.0 (8.3) to 12.7 (17.3) and from 3.8 (7.5) to 5.1 (9.8) in the control group. In S-WMFT, we observed an improvement of 39% in the study group and 25% in the control group. No statistically significant changes were observed between the groups within FMA-UE.
Castelli L. et al., 2025 [25]	*N* = 24 (10 men, 14 women) *N* = 12 (in experimental group) Mean age (SD) 68 in experimental group and 57.58 in control group Adults with ischemic or hemorrhagic stroke within 6–12 months after stroke.	Both groups participated in 45 min physiotherapy sessions three times a week for a period of 4 weeks. Both groups performed exercises aimed at improving hand motor function, and the research group additionally participated in robot-assisted therapy.	FMA-UE (Fugl–Meyer Assesment of the Upper Limb) MAS (Modified Ashworth Scale) MI-UL (Motor Impairment—Upper Limb	Both groups showed statistically significant improvement. In the study group, there was an increase in MI-UL (*p* = 0.002) and FMA-UE (0.002) scores and a decrease in MAS (*p* = 0.009). The control group showed improvement in MI-UL (*p* = 0.002), FMA-UE (0.002), and a decrease in MAS (*p* = 0.024). A comparison of the results of both groups shows that the study group showed greater improvement in MI-UL (*p* < 0.001), FMA-UE (0.002), and a decrease in MAS (P-0.024).
Bayindur O. et al., 2022 [17]	*N* = 37 (21 men, 12 women) *N* = 16 (in experimental group) Mean age (SD) 58 in control group and 55.5 in experimental group. Adults with ischemic or hemorrhagic stroke min 3 months prior	Both groups participated in physiotherapy sessions lasting 3 h, twice a week for a period of 5 weeks. The treatment was aimed at restoring the motor functions of the directly affected upper limb. During each training session, the research group received an additional hour of work with the robot.	FMA-UE (Fugl–Meyer Assesment of the Upper Limb) BBT(Box and Block Test) Nine—Hole Peg Test Jebsen–Taylor Hand Function Test Grip strength Pinch Test	A statistically significant improvement was observed in the results of both groups. When comparing the measurement results, they showed much better results in the study group than in the control group in BBT and Pinch Test measurements (*p* < 0.05).
Li Y. et al., 2024 [11]	*N* = 44 (33 men, 7 women) *N* = 22 (in experimental group) Mean age (SD) 63.7 in control group and 63.6 in experimental group Adults with ischemic or hemorrhagic stroke up to 6 months prior.	Both groups participated in 40 min therapeutic sessions aimed at restoring upper limb function every day for a period of 4 weeks. In addition, the research group participated in 20 min training sessions using a force feedback robot, while the control group participated in 20 min training sessions in which the therapist took over the function of the robot.	FMA-UE (Fugl–Meyer Assesment of the Upper Limb) ARAT (Action Research Arm Test) Grip strength MAS (modified scale) Range of motion	Both groups showed statistically significant improvement in FMA-EU, ARAT, ROM, and grip strength. No significant change in MAS was observed. However, the results of the study group proved to be statistically better (*p* < 0.05).
Lee H.-C. et al., 2021 [14]	*N* = 24 (16 men, 8 women) *N* = 14 (in experimental group) Mean age (SD) 53.5 in control group and 59.56 in experimental group. Adults with ischemic or hemorrhagic stroke min 3 months prior	Both the control and research groups participated in 60 min therapy sessions twice a week for a period of six weeks. Patients in both groups performed the same exercises during the intervention; however, the control group used a robot, while the research group was supervised only by a therapist.	FMA-UE (Fugl–Meyer Assesment of the Upper Limb) BBT (Box and Block Test) EMG MBI (Modified Barthel Index)	Both groups showed improvement in results after completing therapy. However, the study group showed greater improvement than the control group in terms of FMA-UE (*p* = 0.46), BBT, and MBI (*p* = 0.038).

## Data Availability

The data presented in this study are openly available in PubMed, BMJ Journals, Directory of Open Access Journals, Science Direct, Research Gate, and Sage Journal.

## Supplementary Materials

The following supporting information can be downloaded at: https://www.mdpi.com/article/10.3390/jcm15082951/s1, PRISMA Checklist [35].

## Abbreviations

The following abbreviations are used in this manuscript:

EG	Experimental Group
CG	Control Group
FMA-UE	Fugl–Meyer Assessment for Upper Extremity
MAS	Modified Ashworth Scale
DALYs	Disability-Adjusted Life Years
GDP	Gross Domestic Product
ARAT	Action Research Arm Test
BBT	Box and Block Test
ROM	Range of Motion
AROM	Active Range of Motion
RG	Robot Group
WMFT	Wolf Motor Functional Test
NHPT	Nine-Hole Peg Test
BI	Barthel Index
BRS-H	Brunnstrom Recovery Stages for the Hand
MI-UL	Motor Impairment Upper Limb
SULCS	Stroke Upper Limb Capacity
ADL	Activities of Daily Living
MBI	Modified Barthel Index
JTHFT	Jebsen–Taylor Hand Function Test
